# Accessory Spleen Masquerading as an Intrapancreatic Tumor: A Case Report

**DOI:** 10.7759/cureus.24677

**Published:** 2022-05-02

**Authors:** Harrison C Smith, Nandita Kakar, Anthony M Shadid

**Affiliations:** 1 Medical School, Nova Southeastern University Dr. Kiran C. Patel College of Osteopathic Medicine, Fort Lauderdale, USA; 2 Radiology, University of Illinois at Chicago, Peoria, USA

**Keywords:** abdominal pain, nuclear medicine imaging, pancreatic tumor, accessory spleen, ectopic spleen, splenosis

## Abstract

The finding of splenic tissue within the pancreas, also known as splenosis or intrapancreatic accessory spleen (IPAS), is a relatively uncommon condition that presents as an intrapancreatic mass. The discovery of an intrapancreatic mass often prompts a thorough diagnostic workup for a primary pancreatic malignancy, often exposing patients to unnecessary risks associated with invasive testing and even surgery. The benign, asymptomatic nature of this finding places emphasis on utilizing non-invasive techniques for confirmation of the diagnosis, reducing risks of morbidity and mortality in this patient population. Contrast-enhanced computed tomography (CT) and magnetic resonance imaging (MRI) will display near-identical signal intensities (SI) between the spleen and the intrapancreatic mass, as well as identical contrast-enhancement patterns. Nuclear medicine evaluation with Tc-99m heat-damaged red blood cells (HDRBCs) is often used as a confirmatory test and allows for differentiation from malignancies.

## Introduction

The presence of an accessory spleen is the result of the abnormal migration of mesenchymal cells in the dorsal mesogastrium [1]. The majority of identified cases have been localized to the splenic hilum; however, accessory spleens have been discovered in a wide range of anatomic locations, including the pancreas [2]. There is ongoing debate regarding the incidence of the accessory spleen; however, reports estimate its presence in around 10% of the general population [3]. Accessory splenic tissue is detected 13 times more often in females than in males, with a reported mean age of between 20 and 40 years [4]. The intrapancreatic location of an accessory spleen poses a unique diagnostic challenge for physicians, as the possibility of malignancy must be ruled out. We present the case of a 45-year-old male in which an intrapancreatic mass was discovered. With a high index of suspicion, the diagnosis of intrapancreatic accessory spleen (IPAS) was made with non-invasive techniques including computed tomography (CT), magnetic resonance imaging (MRI), and nuclear medicine imaging.

## Case presentation

A 45-year-old male with a past medical history significant for hypertension and hyperlipidemia presented to the emergency department due to one-day history of right lower quadrant pain described as a dull intermittent ache that progressed to a constant stabbing pain in the right lower quadrant that woke him from sleep. He admitted to associated nausea and a single episode of vomiting at home before presenting to the emergency department (ED). At the time of conducting the history and physical, the patient stated that his pain had improved and was rated a 1/10. He also admitted to subjective fever and chills at the time of the interview. He denied any chest pain, shortness of breath, diarrhea, or constipation.

On physical exam, the patient was afebrile and hemodynamically stable. The abdomen was soft but was notable for mild direct right lower quadrant tenderness with no guarding or rebound tenderness. The remainder of the physical exam was unremarkable. Labs drawn in the ED revealed a leukocytosis of 14,400/mm^3^. Attention was turned toward the possibility of an acute inflammatory process such as appendicitis, cholecystitis, or colitis.

**Figure 1:**
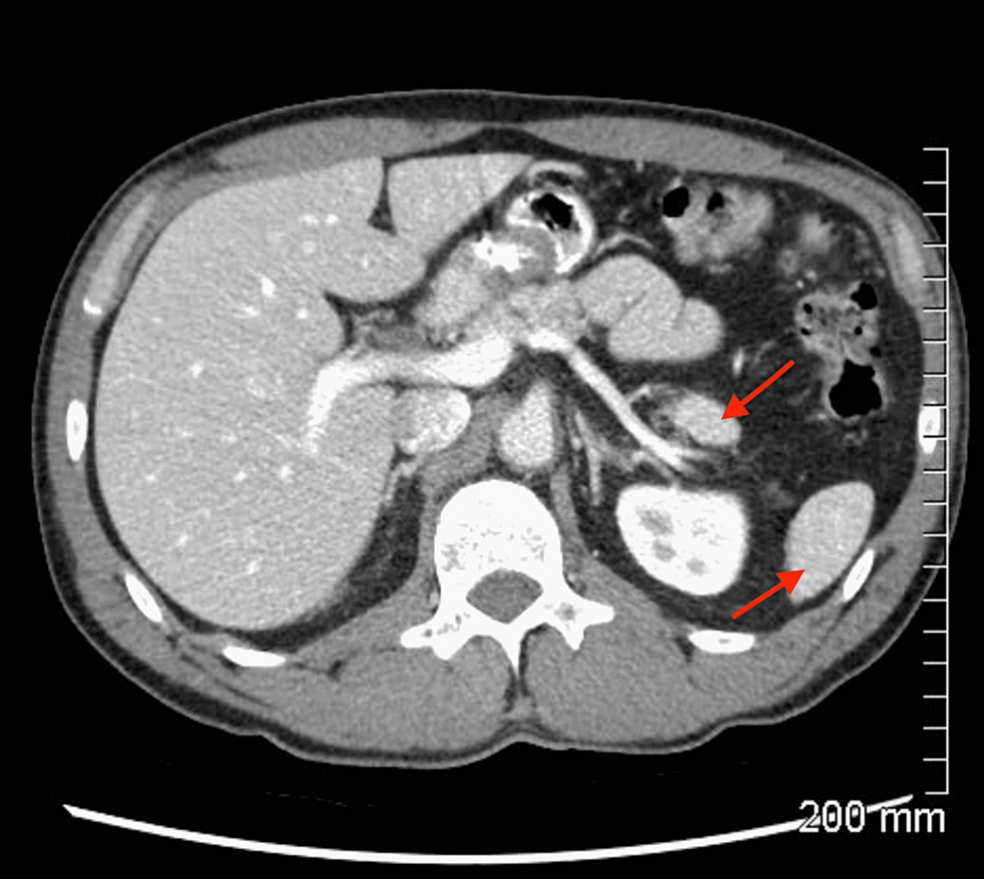
Abdominal CT with contrast showing a hyperintense, enhancing 1.9 cm soft tissue mass within the pancreatic tail distinct from the surrounding parenchyma. Arrows indicate the pancreatic mass and spleen.

A CT of the abdomen and pelvis was performed and revealed a 1.9 cm soft tissue mass in the pancreatic tail (Figure 1). Notably, the mass was morphologically distinct from the remainder of the pancreatic parenchyma, warranting further correlative imaging.

**Figure 2:**
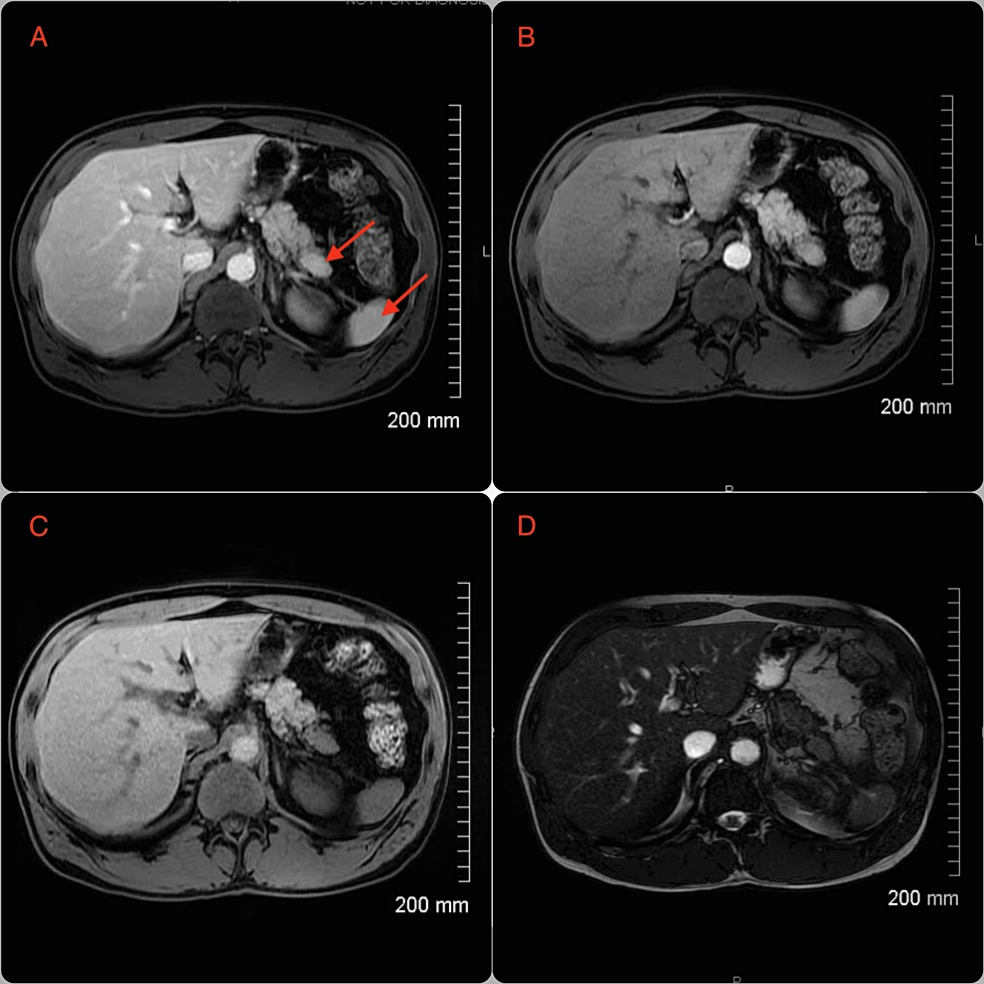
Axial MRI shows a 1.4 cm × 2.3 cm mass in the superior pancreatic tail isointense to the spleen with similar enhancement characteristics. (A) Pre-T1 fat saturated MRI, (B) arterial phase T1 MRI, (C) delayed phase T1 MRI, and (D) T2 MRI.

An MRI of the abdomen with and without IV multihance contrast was performed to further characterize the pancreatic mass. A 1.4 cm × 2.3 cm mass was visualized in the superior pancreatic tail (Figure 2). The mass was hypointense and hyperintense to the adjacent pancreatic parenchyma on T1 and T2-weighted imaging, respectively. Rapid enhancement was noted on postcontrast images. The remainder of the pancreas, including the head and duct, was unremarkable. The initial impression of this lesion led to a recommendation for percutaneous biopsy with interventional radiology.

Upon review of the available imaging, typical enhancement and signal characteristics suggestive of a benign intrapancreatic splenule were noted. Instead of biopsy, evaluation with a nuclear medicine technetium-99m heat-denatured tagged RBC scan for confirmation of this suspicion was recommended.

**Figure 3:**
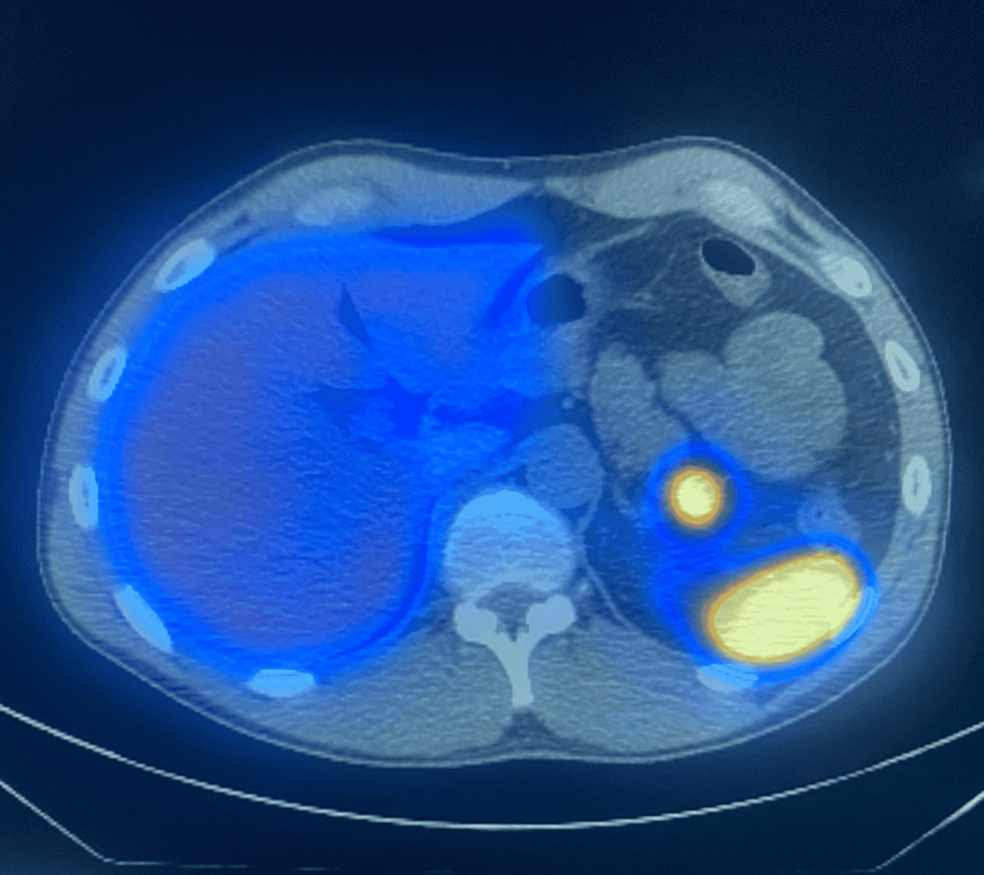
SPECT-CT/fusion imaging demonstrating splenic and ectopic radiotracer uptake.

A 3.1 mCi technetium 99m heat-damaged red blood cell (HDRBC) scan was performed using SPECT-CT and fusion imaging. Findings were significant for focal radiotracer uptake directly corresponding to the soft tissue mass in the pancreatic tail, which was similar in intensity to the spleen (Figure 3). This finding is consistent with the diagnosis of a benign intrapancreatic splenule. No other splenules were visualized.

## Discussion

The spleen is a large encapsulated organ in the left upper quadrant of the abdominal cavity consisting of vascular and lymphoid tissue. Ectopic splenic tissue can be congenital or may be acquired due to trauma. The majority of ectopic spleens are found at the splenic hilum. Cases have also been reported in the pancreas, greater omentum, along the greater curvature of the stomach, as well as the small and large intestines. Splenosis may occur due to autotransplantation of splenic tissue during splenectomy or trauma. Splenosis can present as up to 100 splenic nodules varying in location within the intraperitoneal space, whereas congenital accessory spleens are usually fewer in number, with a maximum of six [4]. The patient, in this case, has no history of trauma or splenectomy, which further suggests a case of undiagnosed intrapancreatic accessory spleen.

Intrapancreatic accessory spleen, also referred to as intrapancreatic splenule, is defined as the presence of ectopic splenic tissue within the pancreatic parenchyma. The ectopic splenic tissue is inherently benign and is a result of a congenital abnormality in the embryological fusion of the spleen [5]. Although the intrapancreatic location of an accessory spleen is relatively rare, an accessory spleen is believed to be present in 10% of the population [6]. IPAS is often clinically silent with no overt symptoms, further lending to the low detection rate. IPAS is often detected incidentally during a workup for other intra-abdominal pathologies. However, many splenules are too small to be detected on imaging.

Pancreatic masses are often initially detected on CT. Visualization of a solitary pancreatic mass on CT, especially when it is an enhancing lesion, has a differential diagnosis including pancreatic neoplasm until proven otherwise. The differential diagnosis of solid pancreatic lesions includes adenocarcinoma, neuroendocrine tumors, pancreatic cystic neoplasms, solid pseudopapillary tumors, pancreatic lymphoma, and metastasis. There have been multiple reports of intra-pancreatic accessory spleens being mistaken for malignancies, which were subsequently either biopsied or surgically resected [7,8]. These invasive procedures are commonly performed due to diagnostic uncertainty based on available radiologic evidence. Considering the risks of morbidity and mortality associated with undergoing these procedures, it is important to keep in mind that the differential for a pancreatic mass is broad, and one should not exclude the possibility of ectopic splenic tissue if imaging findings are supportive of the diagnosis. A high index of suspicion can allow for the diagnosis of IPAS using non-invasive imaging modalities, avoiding the risks of biopsy or surgical resection.

CT findings characteristic of accessory spleens are well-marginated, round masses that are often smaller than 2 cm and enhance homogeneously on contrast-enhanced images [9]. Careful attention should be turned toward the pattern of enhancement, as the pattern will be identical to the enhancement pattern of the spleen. In a study of 180 accessory spleens examined with CT, Mortelé et al. described the possibility of the accessory tissue appearing hypodense compared to the main spleen if the lesion measured less than 1 cm and attributed this finding to a likely result of volume effects [9].

A pancreatic mass found on CT often leads to a follow-up study with MRI for further characterization. Similar to CE-CT imaging, it is important to note the enhancement pattern of the pancreatic mass and assess its similarity to the enhancement pattern of the spleen. When compared to pancreatic parenchyma, IPAS has a relatively low signal intensities (SI) on pre-contrast T1-weighted imaging and a relatively high SI on pre-contrast T2 imaging. This is similar to various hypervascular tumors found in the pancreas [10]. A hallmark of IPAS on MRI imaging is the isointensity with the spleen on T1 and T2-weighted imaging as well as the inhomogenous enhancement pattern on arterial phase images [11]. Neoplastic lesions in the pancreas may have similar SI to the spleen on MRI, posing additional diagnostic uncertainty.

Nuclear medicine testing, using technetium 99m HDRBC followed by multiplanar and SPECT-CT imaging, is a highly specific method used in the detection of splenic tissue. Analogous to how naturally damaged red blood cells are taken up by the spleen and reticuloendothelial system, up to 90% of the injected Tc-99 labeled HDRBCs are taken up by splenic tissue [10]. The relatively poor spatial resolution of SPECT-CT when compared to CT or MRI cross-sectional imaging places particular emphasis on the need to correlate findings from each modality. Perhaps the greatest benefit of the use of nuclear medicine imaging is the differentiation of neuroendocrine tumors and other malignant pancreatic neoplasms, demonstrating its high positive predictive value.

## Conclusions

While ectopic splenic tissue presenting as an accessory spleen is not an uncommon phenomenon, the intrapancreatic location of such masses is seldom reported. They pose a diagnostic challenge when discovered on imaging, as they may be initially suspected to be an intrapancreatic malignancy. Careful consideration of the imaging characteristics of the mass, as well as the utilization of appropriate imaging modalities, can allow for confirmation of the IPAS without invasive procedures or surgical intervention. This is of great potential benefit to the patient, as IPAS is an inherently benign mass, and intervention is unnecessary in an overwhelming majority of circumstances.
